# Legius Syndrome and Inflammatory Bowel Disease: A Pediatric Case Report

**DOI:** 10.7759/cureus.46394

**Published:** 2023-10-03

**Authors:** Filipa Paixao, Luisa Ribeiro, Adriana Costa, Maria Torre, Ana Ventura

**Affiliations:** 1 Pediatrics, Hospital Professor Doutor Fernando Fonseca, Lisbon, PRT

**Keywords:** inflammatory bowel disease, pediatrics, café-au-lait macules, neurofibromatosis, legius syndrome

## Abstract

Legius syndrome (LS) is a rare and underrecognized disorder that is often misdiagnosed as neurofibromatosis type 1 (NF1). It is characterized by café-au-lait macules without the tumoral manifestations of NF1. We report the case of an 11-year-old patient with multiple café-au-lait macules and intertriginous freckling who was admitted for bloody stools, joint pain, and weight loss. His clinical and endoscopic findings were consistent with inflammatory bowel disease (IBD). He also met the clinical diagnostic criteria for NF1 but not for LS. Genetic testing played a pivotal role in the differential diagnosis and revealed a loss-of-function mutation in the *SPRED1 *gene, confirming the diagnosis of LS. This is the first reported case of a patient with IBD and LS. The subtle manifestations of LS make it an underdiagnosed disease, which reduces the likelihood of it being diagnosed in association with other diseases, such as IBD. There are, however, 10 published case reports linking IBD and NF1, and some pathophysiological mechanisms have been proposed. Continued reporting will help clarify the relationship between IBD and RASopathies such as NF1 and LS.

## Introduction

Legius syndrome (LS) is a rare disease that was first described in 2007, with fewer than 300 reported cases to date. It is caused by loss-of-function mutations in the *SPRED1 *gene (15q14). In its non-mutated form, the *SPRED1 *gene works as a negative regulator of the Ras/mitogen-activated protein kinase (MAPK) pathway, inhibiting neurofibromin, the product of the *NF1* gene. Mutations in the *NF1 *gene are a well-established cause of type 1 neurofibromatosis (NF1) [1].

Various allelic variants associated with LS have been described, although no genotype-phenotype correlation has been identified yet. LS follows an autosomal dominant inheritance pattern and affects approximately 1 in 46,000 to 75,000 individuals worldwide, which is significantly less common compared to NF1. The true incidence of LS is unknown and likely underestimated as some LS patients meet the diagnostic criteria for NF1. This overlap can lead to incorrect diagnosis in the absence of genetic testing [1].

Similar to NF1 patients, LS patients typically exhibit café-au-lait macules, intertriginous freckling, and developmental delay. Less frequent manifestations of LS may include macrocephaly, pectus excavatum/carinatum, and lipomas. LS patients typically do not display NF1 tumoral manifestations such as Lisch nodules, optic nerve gliomas, and neurofibromas [1]. It is important to note that there are specific *NF1* gene mutations that are characterized solely by cutaneous manifestations, making genetic testing essential for differential diagnosis [2]. In contrast to NF1, LS is not associated with a well-established risk of malignancy. In 2015, the functional role of the *SPRED1 *gene as a tumor suppressor in acute leukemia was reported for the first time. This may raise the question of whether acute leukemia could be a rare complication of LS [3].

Furthermore, there are a few reported associations between NF1 and inflammatory bowel disease (IBD), but no association between LS and IBD has been made so far. IBD is characterized by chronic intestinal inflammation and includes three main phenotypic subtypes, namely ulcerative colitis (UC), Crohn’s disease (CD), and IBD unclassified. UC is limited to the colon and features continuous inflammation of the superficial colonic mucosa, without granulomas on biopsy. Alternatively, CD can affect the entire gastrointestinal mucosa from the mouth to the anus and features non-continuous transmural inflammation [4]. Although both subtypes can have extraintestinal manifestations, the incidence is highest in CD patients [5]. IBD unclassified is a particular subtype of IBD, where chronic gastrointestinal inflammation is present, with subtle manifestations of UC and CD [4]. The pathogenesis of IBD is multifactorial and is thought to include environmental factors, intestinal microbiota, immune system, and genetic susceptibility. Although there is abundant evidence that genetic syndromes, such as Turner syndrome and Down syndrome, are associated with a higher risk of IBD, the same does not hold true for RASopathies such as NF1 and LS [6].

There are no published case reports linking LS and IBD, and only 10 cases of patients with NF1 and IBD have been reported to date: seven cases of UC and three cases of CD. Five of these were diagnosed in the pediatric age group, of which four had UC and one had CD [6-9]. To date, no causal relationship between these two conditions has been established, although several hypotheses have been proposed. One of the proposed mechanisms linking NF1 and IBD is based on the activation of the Ras/MAPK pathway, which plays a critical role in controlling inflammation and apoptosis. Increased Ras/MAPK in NF1 promotes inflammation, and inhibitors of this pathway have been shown to reduce intestinal inflammation in animal models of IBD [6]. Another mechanism involves increased activation and degranulation of intestinal mast cells, as observed histologically in certain patients with both NF1 and IBD. The release of inflammatory mediators from activated mast cells into the colonic epithelium serves to further aggravate intestinal inflammation [7,8]. These mechanisms may play a role in the increased susceptibility to IBD among patients with NF1 [6,8].

Continued reporting of cases could definitively establish a causal relationship between LS and NF1 and IBD. This report explores the potential relationship between LS and IBD.

## Case presentation

This report presents the clinical details of an 11-year-old male adolescent, born in Brazil, of mixed ethnicity, with no significant medical or surgical history. He attained all appropriate physical and cognitive developmental milestones with no noted delays in his medical history. Throughout his development, his growth percentiles were consistently charted in the 25th percentile, as well as his head circumference charted in the 50th percentile. He presented to our institution with a two-month history of diurnal and nocturnal bloody stools with mucus, rectal tenesmus, and inflammation in both knees and tibiotarsal joints. He had lost 3.5 kg in the previous month and had no history of nonsteroidal anti-inflammatory drug (NSAID) use. This patient was admitted for clinical stabilization and further etiologic investigation, and the diagnosis of IBD was considered. His parents, two-year-old sister, and nine-year-old brother were healthy and none had similar skin findings or a history of IBD.

**Figure 1:**
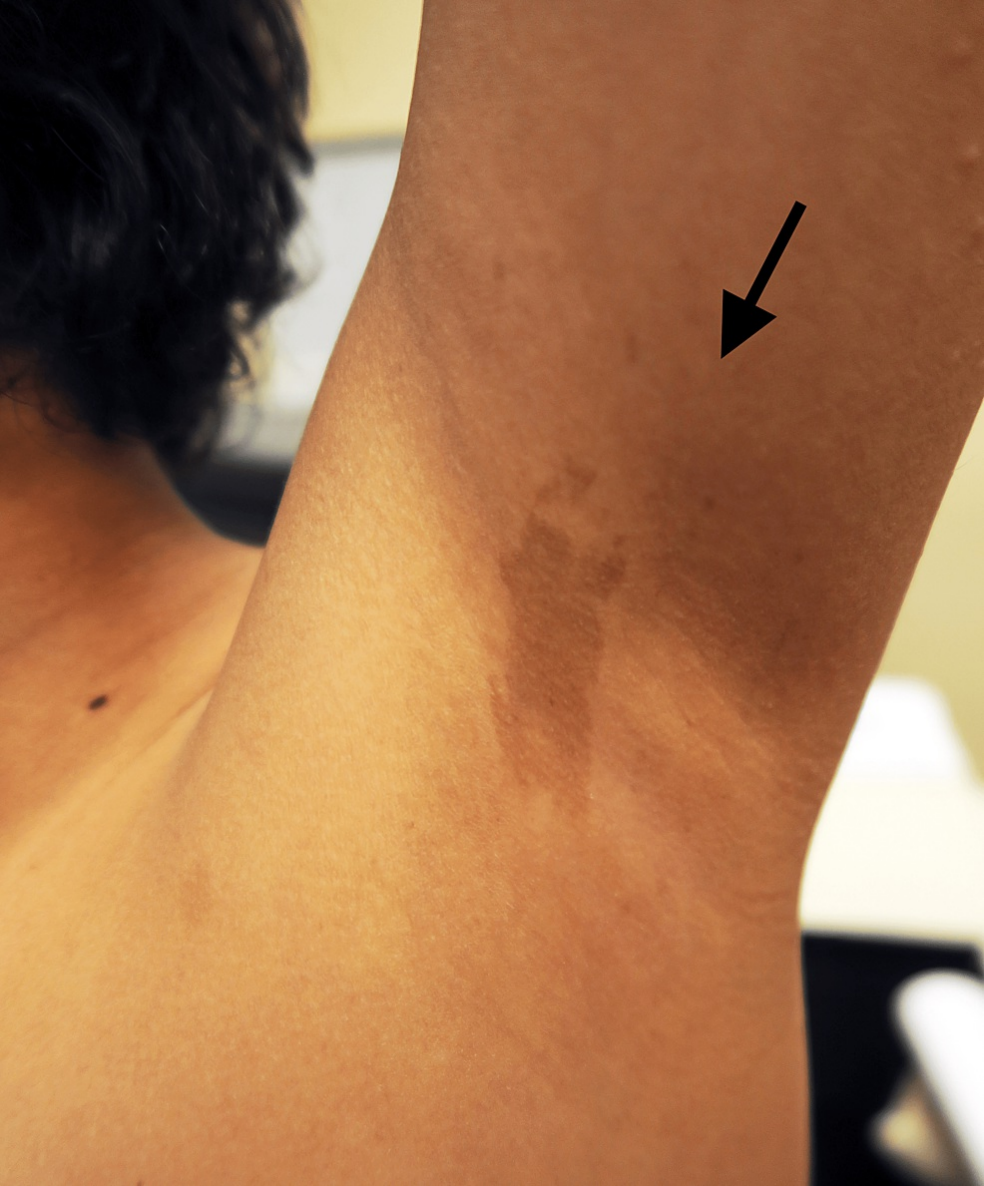
Intertriginous freckling and café-au-lait spot. Left axillary freckling and café-au-lait spot (>10 mm).

**Figure 2:**
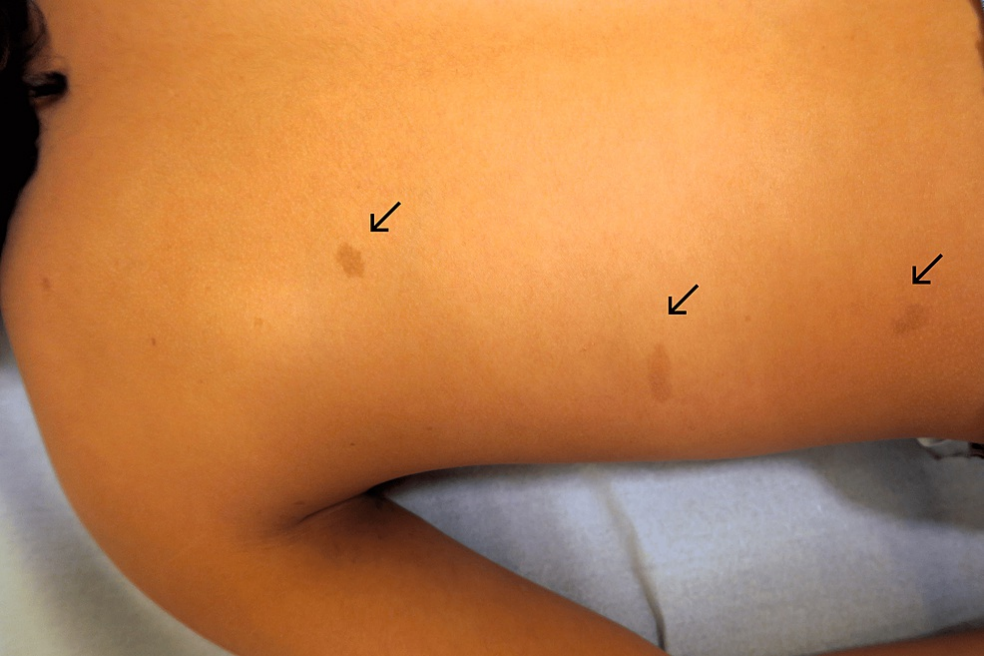
Café-au-lait macules. Three dorsal café-au-lait macules (>5 mm).

On physical examination, skin pallor, intertriginous freckling, and seven café-au-lait macules larger than 5 mm were noted (Figures 1, 2).

The patient was in Tanner stage I. An ophthalmologic examination was conducted to screen for Lisch nodules, which was negative. Additionally, a screening for central nervous system tumors associated with NF1 was performed, including cranial and spinal magnetic resonance imaging, both of which revealed no pathologic findings.

According to the revised diagnostic criteria for NF1, the patient met two key criteria (presence of seven café-au-lait macules larger than 5 mm in a prepubertal child and axillary freckling), resulting in the clinical diagnosis of NF1 [10]. Other clinical conditions characterized by the presence of café-au-lait macules, such as Noonan and Leopard syndromes were considered, but were less likely due to the absence of accompanying organic manifestations. The diagnosis of LS was considered. The diagnostic criteria for LS are met when two of the following criteria are present: (1) five or more café-au-lait macules bilaterally distributed and no other NF1-related diagnostic criteria except for axillary or inguinal freckling; (2) a heterozygous pathogenic (or likely pathogenic) variant in SPRED1 in 100% cells from unaffected tissue; and (3) a parent with the diagnosis of LS by the above criteria [1]. This patient did not meet the clinical diagnostic criteria for LS, as none of his relatives had similar skin findings. The molecular diagnosis was determined through multi-gene panel testing, using next-generation sequencing techniques, and a heterozygous variant was detected in the *SPRED1 *gene: c.304dup, p.(Thr102Asnfs*7).

To establish a diagnosis of IBD, a series of diagnostic tests were conducted. Upon admission, an abdominal ultrasound was performed which revealed left-sided colitis with diffuse mural thickening that was limited to the mucosa and submucosa and enlarged mesenteric lymph nodes. An upper and lower gastrointestinal endoscopy demonstrated antral micro-erosions, without nodular hyperplasia. It also revealed pancolitis with a few erosions and pseudopolyps. No ulcers, fissures, or strictures were observed, and no abnormalities were detected in the perianal region. The histopathological examination exhibited reactive changes in the colonic crypts, accompanied by a mild-to-moderate inflammatory infiltrate, cryptitis, and rare colonic abscesses, without the presence of granulomas. The antrum exhibited non-atrophic chronic gastritis. Microbial testing yielded negative results, including tests for *Helicobacter pylori *and cytomegalovirus.

The patient’s laboratory results (summarized in Table 1) revealed normocytic and hypochromic anemia, elevated systemic inflammatory parameters (C-reactive protein and erythrocyte sedimentation rate), elevated fecal calprotectin and positive results for anti-nuclear antibodies, anti-*Saccharomyces cerevisiae* antibodies (ASCA) (IgG), and perinuclear anti-neutrophil cytoplasmic antibodies (p-ANCA) (IgG).

**Table 1:** Laboratory results at admission. Hb: hemoglobin; MVC: mean corpuscular volume; MCH: mean cell hemoglobin; MCHC: mean cell hemoglobin concentration; CRP: C-reactive protein; ESR: erythrocyte sedimentation rate; ANA: anti-nuclear antibodies; ASCA: anti-saccharomyces cerevisiae antibodies; p-ANCA: perinuclear anti-neutrophil cytoplasmic antibodies; (H): high; (L): low

Parameter	Result	Reference value
Hb (g/dL)	9.4 (L)	11.2–14.5
Hematocrit (%)	31.1 (L)	35.0–44.0
MCV (fL)	81.4	78.0–90.0
MCH (pg)	24.6 (L)	27.0–32.0
MCHC (g/L)	30.2 (L)	32.1–35.9
CRP (mg/dL)	3.5 (H)	<0.50
ESR (mm)	115.0 (H)	<13.0
Albumin (g/dL)	3.8	3.8–5.40
ANA titer	1:160 (H)	Negative
ASCA (IgG)	Positive	Negative
p-ANCA (IgG)	Positive	Negative
Fecal calprotectin (mg/kg)	5,535.0 (H)	<50.0

Ultrasound confirmed synovitis in both tibiotarsal joints, bursitis in the left knee, and enthesitis in the right knee. In addition, a 1 cm diameter oral ulcer developed, and the patient was diagnosed with unclassified IBD (class 2 according to the revised PORTO criteria) [4]. The patient was started on an exclusive oral polymeric diet recommended by the dietitian to reduce gastrointestinal inflammation, but compliance was suboptimal. He was then started on mesalazine (60 mg/kg/day) to induce remission, but due to maintenance of symptoms, prednisone (1 mg/kg/day) was added two weeks later, resulting in a favorable therapeutic outcome. He was asymptomatic at discharge. After discharge, he had a relapse while tapering corticosteroids and was started on azathioprine at 1.5 mg/kg/day. Due to an unfavorable response, he was started on infliximab at 5 mg/kg/dose with a favorable therapeutic outcome.

## Discussion

In the presence of café-au-lait macules, without any other typical manifestations of NF1 such as neurofibromas, optic pathway gliomas, and Lisch nodules, the diagnosis of LS should be considered. Given its autosomal dominant transmission, it is important to obtain the patient’s family history and evaluate the presence of café-au-lait macules in the parents.

The lack of a family history of symptoms of NF1/LS presented a diagnostic challenge in this clinical case. The patient displayed café-au-lait macules, which are typical of both NF1 and LS. Although he met the clinical criteria to be diagnosed with NF1, the same was not true for LS, given his lack of a family history of café au lait macules. Multi-gene panel testing played a pivotal role in the differential diagnosis by enabling the study of different genes linked to a similar clinical presentation. This test revealed a variant in the *SPRED1* gene, known to be pathogenic and associated with LS according to databases. The detected genetic mutation, c.304dup, p.(Thr102Asnfs*7), is a nucleotide duplication that induces a loss-of-function mutation in the *SPRED1 *gene. This mutation impairs the gene’s function as a negative regulator of the Ras/MAPK pathway. Several genetic mutations have been detected; however, no genotype-phenotype correlation has been identified yet [1]. Lastly, the absence of café-au-lait macules in the parents could suggest a de novo diagnosis.

As LS patients do not have NF1 tumor manifestations, the prognosis is largely dependent on the degree of developmental delay, the incidence of pectus excavatum/carinatum, and lipomas. These findings were not present in this patient, highlighting the phenotypic variability of this condition. Awareness of these complications is key to their appropriate monitoring and diagnosis.

To our knowledge, this is the first reported case of a patient with both IBD and LS. However, there are 10 reported cases of patients with NF1 and IBD. Some authors suggest that the increased Ras/MAPK pathway signaling seen in NF1 patients contributes to the development of intestinal inflammation [6]. As LS patients also exhibit Ras/MAPK pathway dysregulation, similar mechanisms may be observed in these patients. Another mechanism involves the activation of intestinal mast cells, as observed histologically in some patients with both NF1 and IBD [8]. However, we did not observe these histologic findings in our patient. There is insufficient evidence linking these diseases. The lower incidence of IBD in LS patients compared to NF1 patients may be attributed to the less frequent diagnosis of LS as opposed to NF1. Further research with larger sample sizes will be necessary to confirm the proposed correlation between LS and IBD.

The diagnostic evaluation of the patient’s IBD involved laboratory tests, imaging studies, endoscopic procedures, and histological analyses. Our patient’s laboratory results revealed normocytic hypochromic anemia, systemic inflammation (elevated C-reactive protein and erythrocyte sedimentation rate), and intestinal inflammation (elevated fecal calprotectin). Fecal calprotectin is indicative of inflammation in the intestinal mucosa but lacks specificity for IBD. Nevertheless, some authors suggest that this marker shows a stronger correlation with the diagnosis of IBD compared to other serum inflammatory markers, such as elevated C-reactive protein or erythrocyte sedimentation rate [4].

The subclassification of IBD was also a diagnostic challenge, as this patient presented with subtle manifestations of both UC and CD. The findings of pancolitis, inflammation limited to the mucosa and submucosa, cryptitis, absence of granulomas, fissures or skip lesions, and positive p-ANCA favor the diagnosis of UC. On the other hand, the findings of gastritis (without a history of NSAID use), vascular congestion and edema of the ileum, and positive ASCA favor the diagnosis of CD. This patient also had synovitis of both tibiotarsal joints, bursitis of the left knee, enthesitis of the right knee, and a 1 cm oral ulcer, all of which are more common in patients with CD [4]. Some studies claim that IBD unclassified is a true intermediate phenotype between CD and UC, but other studies argue that it is only a provisional diagnosis [11]. Adequate long-term follow-up may help further clarify the subclassification and prognosis of this patient’s intestinal disease.

The lack of a clear diagnosis complicated treatment decisions. On admission, this patient was started on an exclusive polymeric diet, which has been shown to induce remission in some patients with CD. Due to suboptimal compliance with this diet, he was started on mesalazine, which has been used to induce remission in UC patients. Due to the maintenance of symptoms, oral corticosteroids were added, which were successful in inducing remission. Oral corticosteroids are used to induce remission in patients with UC and CD. During corticosteroid tapering, the patient relapsed, so azathioprine was added, which is often used in steroid-dependent UC and CD. Due to an unfavorable response, he was also started on infliximab, ultimately with a favorable response.

There are several reasons why long-term follow-up of these patients is essential. Patients should be evaluated in accordance with their growth charts, as chronic diseases such as IBD can delay growth and puberty [12]. IBD patients should also be monitored for colorectal cancer, as a meta-analysis showed a 2.4-fold increase in the relative risk of cancer, primarily associated with gastrointestinal cancer [13]. LS patients should be screened for learning disabilities and developmental delays [1]. In addition, mental health status should be assessed, as the diagnosis of a chronic disease can affect socialization, self-image, school attendance, and academic performance [12]. Awareness of these comorbidities is key to their appropriate monitoring and diagnosis.

## Conclusions

LS is a rare disorder characterized by dysregulation of the Ras/MAPK pathway. Its diagnosis should be considered in the presence of café-au-lait macules and in the absence of NF1 tumor manifestations. Given its autosomal dominant transmission, it is important to evaluate the parents’ skin. The diagnosis of LS in this patient was challenging because he did not meet clinical diagnostic criteria, making multi-gene panel testing an essential tool to confirm the diagnosis.

This is the first reported case of a patient with both LS and IBD. The subtle manifestations of LS make it an underdiagnosed disease, which reduces the likelihood of it being diagnosed in association with other diseases. Further reporting will help to clarify these associations.
